# The comprehensive review of gastric adenocarcinoma and proximal polyposis of the stomach (GAPPS) from diagnosis and treatment

**DOI:** 10.1002/ags3.12708

**Published:** 2023-06-21

**Authors:** Masaaki Iwatsuki, Chihiro Matsumoto, Koshi Mimori, Hideo Baba

**Affiliations:** ^1^ Department of Gastroenterological Surgery, Graduate School of Medical Sciences Kumamoto University Kumamoto Japan; ^2^ Department of Surgery Kyushu University Beppu Hospital Beppu Japan

**Keywords:** endoscopic surveillance, GAPPS, prophylactic gastrectomy

## Abstract

Gastric adenocarcinoma and proximal polyposis of the stomach (GAPPS) was first proposed by Wothley et al. in 2012 as a rare familial gastric cancer syndrome associated with an autosomal dominant form of inheritance. GAPPS is characterized by gastric basal gland polyposis from the hilum to the body of the stomach. Li et al. in 2016 showed that the cause of the disease is a point mutation in the promotor 1B region of the *APC* gene, and genetic testing was used to confirm the diagnosis. If the patient has already developed gastric cancer, treatment should be based on the usual treatment for gastric cancer. If no distant metastases exist, a good prognosis can be expected by performing a total gastrectomy. On the other hand, patients with distant metastasis have a poor prognosis. In the case of dysplasia, prophylactic total gastrectomy is recommended, but because it is highly invasive and postoperative postgastrectomy syndrome must be considered, the decision should be made with careful consideration of the patient's background. Therefore, there are no guidelines for screening for GAPPS, the timing of prophylactic total gastrectomy, or methods of endoscopic surveillance. Because GAPPS is a rare disease, its natural history is still unclear. Further case series are needed to elucidate the molecular biology and clinicopathological features of GAPPS and to establish clinical management, including diagnosis, treatment, and surveillance. In this review, we provide an overview of GAPPS, its clinical management, and its problems, which will be useful for the treatment of GAPPS.

## Graphical abstract

GAPPS is a rare familial gastric cancer syndrome associated with an autosomal dominant form of inheritance, which is characterized by gastric basal gland polyposis from the hilum to the body of the stomach and is associated with the risk of gastric cancer. The mechanisms and natural history of gastric carcinogenesis are still unclear because GAPPS is a rare tumor. Furthermore, the clinical management of GAPPS, including screening, surveillance, and the timing of prophylactic total gastrectomy before the development of gastric cancer, remains unclear.

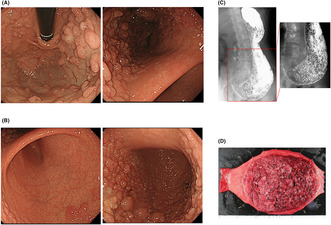

## 1 INTRODUCTION

Gastric cancer (GC) is the fifth most common cancer and the fourth most common cause of cancer death globally.^1^ Several factors reported to have an increased risk of developing GC include *Helicobacter pylori* infection, *Epstein–Barr* virus infection, dietary habits involving salt and salted food intake, alcohol consumption, smoking, and genetic susceptibility.^2^ Approximately 10% of all GC cases show familial aggregation and 1%–3% of patients with GC have germline mutations.^3^

Hereditary diffuse gastric cancer (HDGC), which is well‐known for hereditary GC, was reported in 1998.^4^ Most confirmed HDGC cases are caused by inactivating germline mutations in the *CDH1* tumor suppressor gene, which encodes E‐cadherin, a transmembrane protein that is localized to the adherence junctions in epithelial tissues and has functions in cell‐to‐cell adhesion. The pathogenesis of HDGC has been clarified by the results of many clinical and basic studies to date and has already been published as a medical guideline, establishing clinical indices for diagnosis, treatment, and surveillance.^3^

Gastric adenocarcinoma and proximal polyposis of the stomach (GAPPS) is a new hereditary gastrointestinal (GI) polyposis clinically characterized by Worthley et al.^5^ in 2012. Furthermore, in 2016, Li et al.^6^ reported that the cause of GAPPS is a point mutation in the promoter 1B region of the *APC* gene, and genetic diagnosis has shown an important role in the diagnosis of GAPPS. With the spread of the disease concept, the number of reports of GAPPS has been gradually increasing. GAPPS is diagnosed by genetic diagnosis before the onset of GC, and cases of prophylactic total gastrectomy are increasing. Although surgical techniques have improved, the postoperative complication rate and mortality rate of total gastrectomy have remained high over the past decade.^7^

The mechanisms and natural history of gastric carcinogenesis are still unclear because GAPPS is a rare tumor. Furthermore, the clinical management of GAPPS, including screening, surveillance, and the timing of prophylactic total gastrectomy before the development of gastric cancer, remains unclear. This review will summarize the clinical management of GAPPS in terms of diagnosis, treatment, and surveillance and will hopefully be useful in the practice of GAPPS, which is expected to increase in number in the future.

## 2 DIAGNOSIS

**FIGURE 1:**
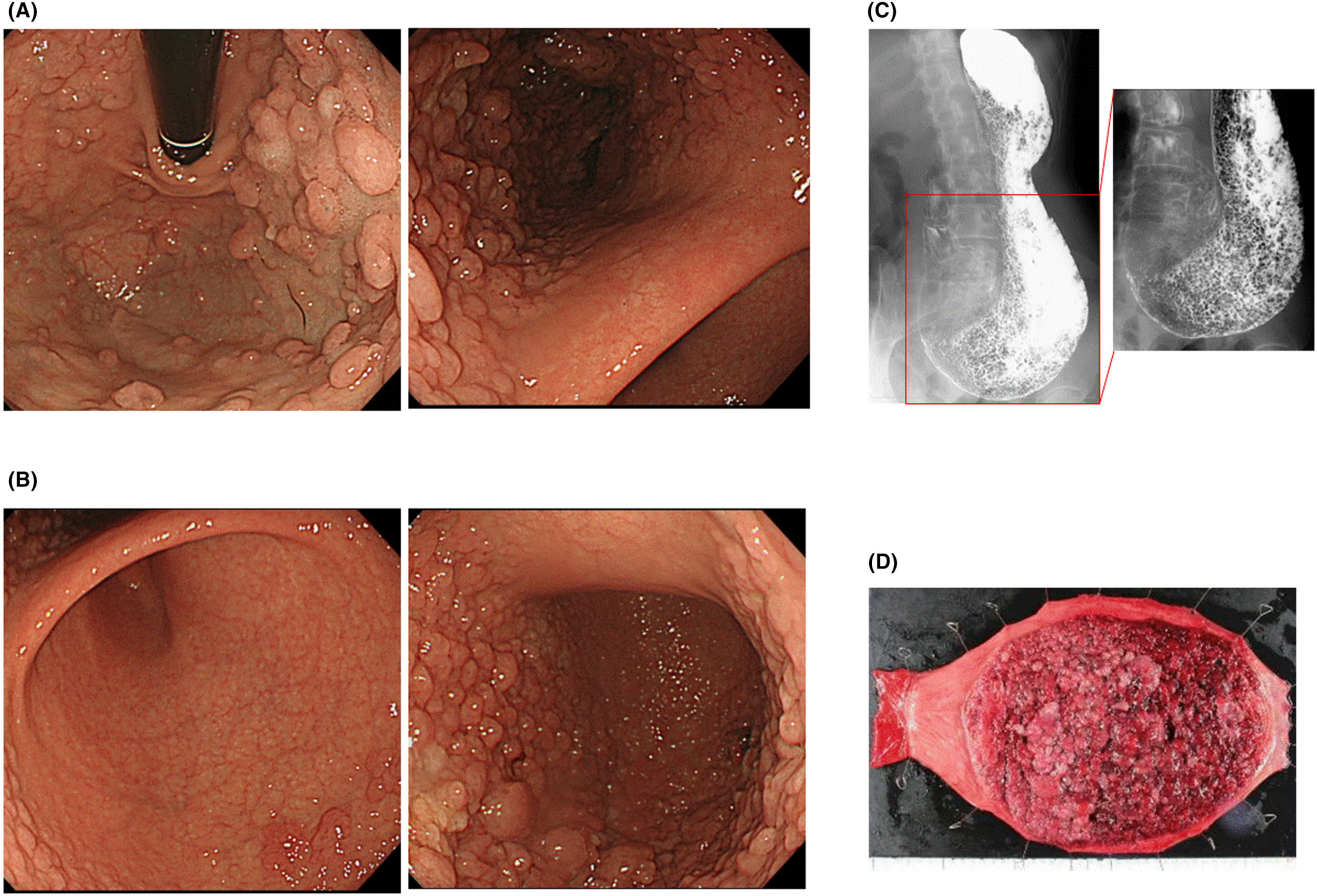
A representative case GAPPS: Fundic gland polyps carpeting the gastric body including cardia, fundus, and body, sparing lesser curve by retroflection view, high‐definition gastroscopy, white‐light imaging (A), uninvolved antrum and pyrolus (B), upper GI examination by barium contrast (C), macroscopic appearance of GAPPS resected specimens (D).

The first paper reporting GAPPS proposed the following clinical diagnostic criteria: (1) gastric polyps restricted to the gastric body and fundus with no duodenal or colorectal polyposis; (2) index case: >100 polyps carpeting the proximal stomach, first‐degree relative of another case: >30 polyps; (3) predominantly fundic gland polyps (FGPs), some with regions of dysplasia (or a family member with either dysplastic FGPs or gastric adenocarcinoma); (4) autosomal pattern of inheritance; (5) exclusion of other gastric polyposis syndromes and the use of proton pump inhibitors (PPIs).^5^ Endoscopic findings of massive gastric polyposis with suspected GAPPS may resemble polyposis as part of other gastric or gastrointestinal polyposis syndromes. The entire fundic gland area from the fundus to the middle of the body is densely populated with “carpet‐like” FGPs several millimeters in size. As shown in Figure 1, the characteristic finding that FGP is not present in the antrum on upper GI endoscopy is a point that can facilitate differential diagnosis to other GI polyposis syndromes such as MUTYH‐associated polyposis, Peutz–Jeghers syndrome, and familial adenomatous polyposis (FAP). However, GAPPS is also characterized by the lack of characteristic findings in endoscopic morphology, color tone, by NBI imaging. Therefore, FGPs cannot be diagnosed by macroscopic findings alone due to the similarity with other GI polyposis syndromes. Previously, diagnosis of GAPPS largely depend on clinical and endoscopic findings as described above. Multi‐cancer gene panels for genetic testing are useful to rule out germline variants associated with other gastric polyposis syndromes, which include the above polyposis‐disposition genes and known hereditary gastric cancer predisposition syndromes. Notably, germline point mutations in the *APC* promoter 1B were recently identified and co‐segregated with GAPPS in six families (one Australian and five North American families).^6^ Patients who meet current clinical criteria for GAPPS are recommended to get genetic testing for point mutations in the *APC* 1B promoter. Some variants of *APC* 1B promoter, including c.‐191T>C, c.‐192A>C, c.‐195A>C, c.‐191T>G, and c.‐125delA were identified.^6^, ^8^, ^9^, ^10^ However, these variants are not thought to reflect the GAPPS phenotype.

### 2.1 Pathological features

**FIGURE 2:**
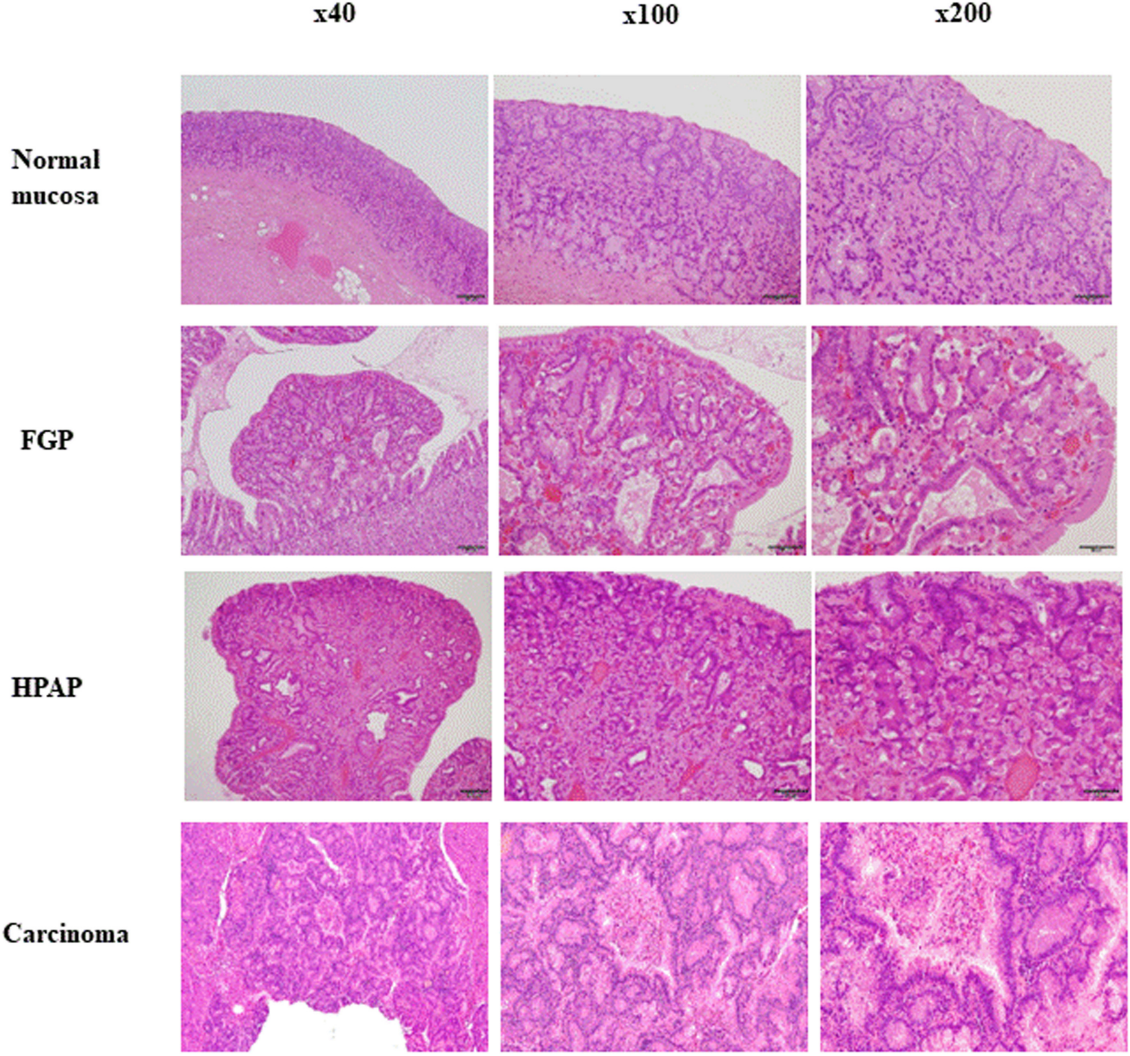
Hematoxylin and eosin (H.E.) section of gastric lesions. Normal mucosa, FGPs contain fundic gland hyperplasia and dilated foveolar epithelium, poloid lesions consistent with hyperproliferative aberrant pits (HPAP), and carcinoma (×40, ×100 and ×200 original magnification).

Boer et al.^11^ examined the detailed pathological analysis of 51 endoscopic biopsies and five gastrectomy specimens from 25 patients defined as GAPPS family members. Boer et al. proposed a novel pathological feature, defined as “hyperproliferative aberrant pits” (HPAP), which are accompanied by elongation, irregularity, dilation, and branching of the pits, lined by foveolar epithelium imparting an appearance of “inverted” foveolar hyperplasia (Figure 2). HPAP presents the disorganized hyper‐proliferation of oxyntic glands of the gastric mucosa around gastric pits, which give rise to polypoid lesions. HPAP is observed to be the most frequent and most early pathological abnormal finding. Furthermore, a wider spectrum of pathological findings with GAPPS was reported compared to previous reports. The dysplasia is a gastric phenotype, and the subsequent adenocarcinoma may follow the gastric pathway of carcinogenesis. Immunohistochemical profiles of FGP with multifocal “flat” dysplasia, and gastric adenomatous polyps, were associated with gastric adenocarcinoma, particularly with the gastric lineage marker MUC5AC shared by both premalignant lesions and adenocarcinoma. The finding was an accompanying neoplastic component in the form of a dysplasia‐adenomatous‐adenocarcinoma sequence in the development of the malignant phenotype of GAPPS. Most recent studies compared a set of polyps from non‐syndromic patients to syndromic FGPs such as FAP and GAPPS and evaluated histological features related to distinct subtypes of FGPs.^12^ Although there are slight differences between FAP‐associated or GAPPS‐related FGP and non‐syndromic FGPs, most have similar histologic features.

## 3 TREATMENT

If GAPPS has already developed GC, proceed with treatment in the same manner as for general gastric cancer. On the other hand, if not already developed with GC, current guidelines on endoscopic surveillance or timing of prophylactic gastrectomy largely depend on expert opinions due to rare diseases.

### 3.1 Endoscopic surveillance

Upper GI endoscopy is useful for the early detection of GAPPS, and endoscopic biopsy at regular intervals is necessary to prevent the development of GC. However, some previous reports questioned prolonged endoscopic surveillance in patients with GAPPS.^10^, ^13^ One case of rapid progression to GC with distant metastasis was reported despite frequent endoscopic surveillance.^10^ In our case, the rapid progression to GC with distant metastasis was observed in a brief period of 10 months.^14^ The micropapillary component was observed in the biopsy specimens, although the proportion was small. Furthermore, sampling in cases of polyposis, which includes FGP with different degrees of dysplasia, is challenging.^13^ Clinically, upon adequate sampling by endoscopic biopsy, we face hundreds of heterogeneous polyp lesions that may include FGPs with varying degrees of dysplasia, adenoma, or mixed‐histology polyps, raising clinical concerns. The timing of endoscopic surveillance initiation also remains controversial, particularly in young patients. A recent report suggested that the first‐degree relatives of patients with GAPPS with a proven *APC* mutation should start undergoing endoscopic surveillance at 15 years of age.^15^ The interval or starting time of endoscopic surveillance should be flexible according to the disease condition.

### 3.2 Prophylactic gastrectomy

Total gastrectomy with lymph node dissection is required if GAPPS has already developed GC without distant metastasis. Table 1 summarizes the cases of therapeutic and prophylactic gastrectomy for GAPPS.^5^, ^8^, ^10^, ^14^, ^16^, ^17^, ^18^, ^19^, ^20^, ^21^, ^22^ On the other hand, if not already developed with GC, prophylactic total gastrectomy for GAPPS must be considered very carefully, with caution, and with a fully individualized approach. Repak et al.^10^ stated that patients who fulfill the original GAPPS criteria and those with FGP progression to dysplasia should be tested for genetic mutations so that prophylactic gastrectomy can be performed appropriately. Furthermore, Foretova et al.^19^ recommended prophylactic total gastrectomy for cases with progressive massive stomach polyposis even without dysplasia. Table 1 summarized the cases who underwent prophylactic total gastrectomy for GAPPS.^8^, ^14^, ^16^, ^19^, ^20^, ^21^, ^22^ Based on these previous reports, the algorithm for GAPPS family members with or without mutation of the 1B promoter of the *APC* gene is proposed.^15^ In early reports, the death of a family member was the main reason for surgery without a genetic test, but in recent reports, preoperative genetic tests are performed to diagnose GAPPS, and prophylactic total gastrectomy has been performed.^14^, ^16^, ^19^, ^20^, ^21^, ^22^ With the spread of minimally invasive surgery, laparoscopic or robotic‐assisted total gastrectomy has become popular.^14^, ^16^, ^21^ Iwaoka et al.^16^ reported from their experience with four GAPPS cases treated by robotic total gastrectomy that there were a large number of polyps, but there was no problem in grasping the stomach wall with forceps. However, total gastrectomy is a complex procedure with a significant risk of postoperative morbidity and mortality.^7^, ^23^ Early complications after total gastrectomy, such as esophagojejunal anastomotic leakage, duodenal stump leakage, and pancreatic fistula related to lymphadenectomy, can be fatal. Furthermore, late postoperative complications, including anastomotic strictures, bile reflux, metabolic bone disease, and anemia can have a significant subsequent impact on quality of life. Therefore, it is important to carefully assess the condition and risks of each patient. Grossman et al.^20^ reported a 10‐year‐old boy with low‐grade dysplasia who underwent prophylactic gastrectomy after a genetic diagnosis. In particular, the impact of prophylactic total gastrectomy on the subsequent growth of pediatric patients needs to be considered.

**TABLE 1:** Cases of gastrectomy for GAPPS.

No.	Author, year [ref.No.]	Age	Gender	Follow‐up period	Indication	Genetic diagnosis	Approach	Procedure
Therapeutic gastrectomy								
1	Worthley et al., 2012 [5]	46	N/A	N/A	N/A	N/A	N/A	Total gastrectomy
		53	N/A	N/A	N/A	N/A	N/A	Total gastrectomy
		56	N/A	N/A	N/A	N/A	N/A	Total gastrectomy
		37	N/A	N/A	N/A	N/A	N/A	Total gastrectomy
		41	N/A	N/A	N/A	N/A	N/A	Total gastrectomy
		31	N/A	N/A	N/A	N/A	N/A	Total gastrectomy
		24	N/A	N/A	N/A	N/A	N/A	Total gastrectomy
		18	N/A	N/A	N/A	N/A	N/A	Total gastrectomy
		58	N/A	N/A	N/A	N/A	N/A	Total gastrectomy
		33	N/A	N/A	N/A	N/A	N/A	Total gastrectomy
2	Yanaru‐Fujisawa et al., 2012 [18]	56	F	<1 year	Maligancy	N/A	N/A	Total gastrectomy
3	Kanemitsu et al., 2020 [17]	39	F	1 year	Maligancy	224T>C	Laparoscopic	Total gastrectomy
		37	F	1 year	Maligancy	224T>C	Laparoscopic	Total gastrectomy
		24	F	<1 year	Maligancy	224T>C	Laparoscopic	Total gastrectomy
		57	M	<1 year	Maligancy	224T>C	Laparoscopic	Total gastrectomy
4	Iwakawa et al., 2022 [16]	30s	F	N/A	Maligancy	N/A	Robotic‐assisted	Total gastrectomy
		40s	F	N/A	Maligancy	N/A	Robotic‐assisted	Total gastrectomy
Prophylactic gastrectomy								
1	Worthley et al., 2012 [5]	33	N/A	2 years	N/A	N/A	N/A	Total gastrectomy
		34	N/A	N/A	N/A	N/A	N/A	Total gastrectomy
2	Repak et al., 2016 [10]	23	F	3 years	Family's death due to gastric cancer	N/A	N/A	Total gastrectomy
		30	F	3 years	Family's death due to gastric cancer	N/A	N/A	Total gastrectomy
3	Beer et al., 2017 [8]	38	F	10 years	High‐grade dysplasia	N/A	N/A	N/A
4	Foretova et al., 2019 [19]	29	F	<1 year	Family's death due to gastric cancer	N/A	N/A	N/A
		34	F	<1 year	Family's death due to gastric cancer	N/A	N/A	N/A
		42	F	1 year	Genetic diagnosis	N/A	N/A	N/A
		50	M	<1 year	Genetic diagnosis	N/A	N/A	N/A
		51	F	<1 year	Family's death due to gastric cancer	N/A	N/A	N/A
		65	F	<1 year	Genetic diagnosis	N/A	N/A	N/A
		27	M	<1 year	Genetic diagnosis	N/A	N/A	N/A
		44	M	10 years	Genetic diagnosis	N/A	N/A	N/A
5	Kunovsky et al., 2019 [21]	43	F	<1 year	Genetic diagnosis	N/A	Laparoscopic	Total gastrectomy
		50	M	<1 year	Genetic diagnosis	N/A	Laparoscopic	Total gastrectomy
6	Matsumoto et al., 2021 [14]	43	M	<1 year	Genetic diagnosis	c.‐224T>C	Laparoscopic	Total gastrectomy
		45	M	<1 year	Genetic diagnosis	c.‐224T>C	Laparoscopic	Total gastrectomy
7	Grossman et al., 2021 [20]	10	M	2 years	Genetic diagnosis	c.‐195A>C	N/A	Total gastrectomy
8	Iwakawa et al., 2022 [16]	40s	F	<1 year	Genetic diagnosis	N/A	Robotic‐assisted	Total gastrectomy
		20s	F	<1 year	Genetic diagnosis	N/A	robotic‐assisted	total gastrectomy
9	Salami et al., 2022 [22]	37	M	<1 year	Genetic diagnosis	c.‐191T>C	Open	Total gastrectomy
		36	M	<1 year	Genetic diagnosis	c.‐191T>C	N/A	Total gastrectomy

Abbreviation: N/A, not available.

### 3.3 Palliative endoscopic resection

Ako et al.^24^ report a case of GAPPS occurring with ball valve syndrome. The patient underwent palliative endoscopic mucosal resection for pyloric gland adenoma and showed no further symptoms or recurrence. Although endoscopic resection is the preferred procedure in terms of organ preservation, long‐term surveillance is required to confirm whether endoscopic treatment is acceptable for GAPPS in cases without curative total gastrectomy. Endoscopic resection may be an option for patients who are not fully fit for surgery or refuse gastrectomy.

## 4 PROGNOSIS

Patients who undergo total gastrectomy with or without GC have a better prognosis, whereas patients with the rapid progression to GC with distant metastasis have a worse prognosis. A 41‐year‐old woman who had been admitted to another hospital for FGP without any symptoms and a rapid progression to gastric adenocarcinoma with multiple liver metastases was observed in a brief period of 10 months and died a year after diagnosis.^14^ Repak et al.^10^ described as a part of their European family a 54‐year‐old index proband with GAPPS who was diagnosed after a 19‐month endoscopic surveillance with liver metastasis and who died shortly thereafter. Worthley et al.^5^ described a 33‐ and 48‐year‐old relative of the initial proband in their GAPPS family who died from metastatic intestinal‐type GC after being diagnosed with GAPPS previously and had undergone a period of prior endoscopic observation. A 48‐year‐old female diagnosed as GAPPS genetically with multiple liver metastases finally died on day 223 after hospitalization despite intensive systemic chemotherapy.^9^ Based on these previous reports, once the disease progresses to distant metastases, the prognosis is very poor. Although the prognosis of GAPPS is highly variable with several phenotypic variations noted in the age of onset, penetrance, and degree of dysplasia, early diagnosis is needed if GAPPS is clinically suspected, leading to appropriate treatment strategy.

## 5 CLINICAL ISSUES

Gastric polyposis restricted to the gastric body and fundus is suspected to be caused by GAPPS, and detailed family history should be obtained to improve the prognosis through diagnosis and early detection in close relatives. However, the problem with GAPPS is that its natural history is not clear and there is insufficient evidence since the disease concept has not yet been clarified and reports are still rare. In addition, because it is a hereditary disease, genetic counseling is essential. There are many problems to be solved, such as whether or not to perform genetic testing on patients themselves, whether or not to perform genetic testing on close relatives when GAPPS is diagnosed, whether or not it is advisable to test minors, and how to treat patients after diagnosis.

Furthermore, GAPPS clinically has several phenotypic variations, including age of onset, penetrance, or degree of dysplasia, inter‐familial heterogeneity by promoter variants (promotor variants: c.‐125 delA, c.‐191T>C, c.‐192A>G, c.‐195A>C), inter‐individual heterogeneity (frequency and type of second hit mutation, actionable mutation or gene alteration), intra‐tumor heterogeneity such as FGPs, dysplasia, adenoma or carcinoma even within the same tumor, and inter‐tumor heterogeneity between primary and metastatic sites even within the same individual. It is not clear at this time whether it is related to invasive metastasis or invasion metastasis. Recent molecular biological and pathological studies may provide some explanation for the heterogeneity of the clinical phenotype of GAPPS. Therefore, heterogeneity among individuals within GAPPS may require an individualized approach to at‐risk and phenotypically affected families.

## 6 PERSPECTIVE

As mentioned above, the molecular biological mechanism of GAPPS remains unclear. Taniyama et al.^25^ compared pathologically adenomas that remained as adenomas for a long time and those that progressed to adenocarcinoma in a short time was and identified risk factors for adenomas that progressed to adenocarcinoma in a short time or those that simultaneously harbored adenocarcinoma. They concluded that tumor size and the presence of depressed lesions in adenomas may suggest that they have progressed to adenocarcinoma in a short time at the gross level. Jung et al.^26^ also performed whole exome sequence analysis on synchronous pairs 15 of attached gastric adenoma and GC; the preferred sequence of mutational events during gastric adenoma to GC progression may be more context‐dependent than colon adenoma progression. The molecular biological mechanism of HDGC was elucidated, the disease concept became popular, and cases were accumulated. As a result, its natural history, diagnostic methods, and treatment strategies were established, leading to the creation of guidelines.^3^ Further molecular analysis helps us to define HDGC syndrome by mutations in CDH1 and closely related genes, rather than through clinical criteria that capture families with heterogeneous susceptibility profiles.^27^ Like HDGC, the molecular biological mechanism of GAPPS will be clarified in the near future, and it is expected that it will be applied to diagnosis and treatment.

Familial adenomatous polyposis is a genetic disorder that closely resembles GAPPS, and prophylactic total colectomy is performed. However, after total colectomy, the patient's quality of life is often greatly reduced due to frequent diarrhea, dehydration, bowel obstruction, anal dysfunction, and development of intraperitoneal desmoid tumors. A recent clinical study showed that low‐dose aspirin safely suppressed the recurrence of colorectal polyps larger than 5 mm in Japanese patients with FAP.^28^ Since FAP and GAPPS have similar pathologies, if the molecular biological characteristics of GAPPS can be elucidated in the future, a therapeutic strategy called chemoprevention may be established for GAPPS.

## 7 CONCLUSION

Although GAPPS is a rare disease, when we find gastric polyposis confined to the gastric hilum, it is easy to suspect this disease because of the clinical features of GAPPS. At that time, a detailed family history is expected to improve the prognosis by diagnosis and early detection of close relatives. However, since the concept of the disease and the responsible gene have not been reported for some time, the number of cases is small and evidence is lacking. In the future, it is important to systematically accumulate cases by establishing a global registry system.

## FUNDING INFORMATION

The authors declared that no grants were involved in supporting this study.

## CONFLICT OF INTEREST STATEMENT

Author K.M and H.B are editorial board member of *Annals of Gastroenterological Surgery*.

## ETHICS STATEMENTS

Approval of the research protocol: N/A.

Informed Consent: N/A.

Registry and the Registration No. of the study/trial: N/A.

Animal Studies: N/A.
